# Radical Cure of Hydrocele

**Published:** 1845-06

**Authors:** 


*Radical cure of Hydrocele.*—Dr. A. A. Harvey has successfully employed the following plan of curing hydrocele:—First, discharge the fluid with a trochar, or pocket lancet, and then apply a warm vinegar poultice all over the scrotum, in order to bring on inflammation, which generally takes place in a few hours, and becomes painful. When sufficient inflammation has been excited, remove the vinegar poultice, and apply a bread and milk poultice. In a short time, the pain and inflammation generally subside, and the cure is complete. Give a few smart doses of purgative medicine.—*Lancet*, Jan. 11, 1845,

